# Randomised controlled field trial to assess the safety and efficacy of a killed autologous vaccine against *Streptococcus dysgalactiae* subspecies *dysgalactiae* in a sheep flock

**DOI:** 10.1002/vetr.5667

**Published:** 2025-10-10

**Authors:** Joseph W. Angell, Naseem Ahmed, Louise P. Jackson, Konstantina Kasiora, Keith Ballingall, Jennifer S. Duncan

**Affiliations:** ^1^ Department of Research and Innovation Wern Vets Ruthin UK; ^2^ Department of Livestock and One Health Institute of Infection, Veterinary and Ecological Sciences, University of Liverpool Neston UK; ^3^ Moredun Research Institute Penicuik UK

**Keywords:** efficacy, joint‐ill, lambs, neonatal infectious arthritis, *Streptococcus dysgalactiae* subspecies *dysgalactiae*, vaccine

## Abstract

**Introduction:**

Joint‐ill in neonatal lambs, primarily caused by *Streptococcus dysgalactiae* subspecies *dysgalactiae* (SDSD), results in increased mortality and morbidity. An effective vaccine is needed to prevent the disease.

**Methods:**

A blinded randomised controlled trial was carried out using an autologous SDSD vaccine in a commercial sheep flock in the UK. Two doses of vaccine were administered to 481 pregnant ewes, with 509 ewes left unvaccinated. The SDSD antibody titres of the ewes and their lambs were then measured. Any adverse effects or cases of joint‐ill were also reported.

**Results:**

Ten cases of joint‐ill occurred, with five of these lambs born to vaccinated ewes and five to unvaccinated ewes. Serum from 85 vaccinated ewes and 88 control ewes was analysed using an SDSD antibody ELISA, with higher titres found in vaccinated ewes. Higher SDSD titres were observed in lambs from vaccinated ewes (*n* = 87) than in lambs from unvaccinated ewes (*n* = 91). No differences were detected between colostrum samples from vaccinated and unvaccinated ewes. No vaccination‐associated adverse effects were detected.

**Limitations:**

Randomisation was effective; however, ELISA data were limited to mainly crossbred ewes, reducing the power of the breed comparisons.

**Conclusions:**

The vaccine had no effect in reducing the number of lambs with joint‐ill born to vaccinated ewes compared with unvaccinated ewes. However, vaccinating ewes did have a positive effect on their serum SDSD antibody concentrations and those of their lambs.

## INTRODUCTION

Neonatal infectious arthritis (NIA), or ‘joint‐ill’, is a bacterial disease of one or more joints of young lambs, most commonly caused by *Streptococcus dysgalactiae* subspecies *dysgalactiae* (SDSD).^1^, ^2^, ^3^, ^4^ It typically occurs within the first month of life and causes poor animal welfare, including increased mortality. It also negatively effects production, with affected lambs having reduced growth rates.^2^ Disease control often involves prophylactic and metaphylactic antibiotic use, with a variety of products reported, including those from the European Medicines Agency category D and C lists.^2^, ^5^, ^6^, ^7^

The route of entry of SDSD into lambs is not well elucidated, and more than one route may be involved, with oral, navel, ear tag wounds, castration and tail docking wounds all potential routes of entry.^1^, ^3^, ^4^, ^5^, ^8^ On endemic farms, SDSD may be found ubiquitously in the farm environment, including in lambing pens and fields.^9^, ^10^ The pathogen can survive for long periods of time in straw bedding and soil,^8^, ^9^ providing ample opportunity for opportunistic infection in lambs. Ewes may also be direct sources of infection via contaminated teats, milk, vaginal tract or wool.^3^, ^4^, ^8^

The incubation period of SDSD is not well understood, although there is some evidence to suggest that a period of between 24 and 96 hours could be expected.^11^ As the time of onset of clinical signs can vary from farm to farm, this incubation information can assist clinicians in working out the likely source and route of infection, although it does not rule out multiple sources and routes. It is rare for older lambs and adult sheep to develop clinical signs of infectious polyarthritis despite evidence that a proportion may be carrying the pathogen, especially in the digestive tract,^10^ and early work demonstrated the ability of blood from recovered lambs to prevent the growth of SDSD under laboratory conditions, implying an effective immunological response.^12^ Given these findings, a vaccine administered to ewes before lambing could be effective in preventing disease through the transfer of SDSD‐specific antibodies via the colostrum. The expected benefits of a successful vaccine are obvious, with an expected reduction in morbidity and mortality, improved production outcomes (especially growth rates) and a reduction in responsive and preventive antibiotic use. Autogenous killed vaccines to SDSD have been used on affected farms with reported positive results, although robust clinical trials assessing their safety and efficacy are lacking.^10^

Therefore, the aim of this study was to investigate the clinical and immunological effects of a whole‐cell killed autogenous vaccine against two SDSD isolates from the same farm in a blinded, randomised controlled trial.

## METHODS

### Farm background

The study farm was located in the UK and comprised a single commercial sheep flock of 990 ewes. The ewes were broadly kept in two groups: Welsh ewes (*n* = 368) and crossbred ewes (*n* = 622), which were predominantly Welsh mules. Both groups were lambed from the middle of March, with the crossbred ewes lambed indoors on straw‐bedded yards. These ewes were housed for 6 weeks before lambing, and ewes and lambs were housed for 72 hours after lambing before turning out to pasture. The Welsh ewes were lambed outside at pasture, and were only brought inside after lambing if the ewe or lambs required any specific intervention, for example, treatment for infection. In the year preceding the study, approximately 70 lambs were affected with joint‐ill, with similar numbers in other years.

### The vaccine

The vaccine was manufactured by Ridgeway Biologicals FL09/11/22b (CEVA). Two SDSD isolates from the same farm were obtained from infected lamb joints from the previous lambing period. The vaccine was manufactured by collecting a suspension of the SDSD bacteria, inactivating with formaldehyde and emulsifying with synthetic oil, before being preserved with thiomersal. A 2 mL dose was to be injected intramuscularly twice, 3–4 weeks apart, with the second dose given 3–4 weeks before lambing.

### Safety study

Before commencement of the trial, a safety study was carried out. This occurred in three phases:

1. Two healthy non‐pregnant ewes were injected with 2× 2 mL of the vaccine, given intramuscularly at different sites (the gluteals and neck musculature), and then monitored closely for 1 hour, including visual, behavioural and temperature checks. These ewes were then monitored once daily with a visual and temperature check for 7 consecutive days. Any alterations in temperature or injection site reactions were monitored and recorded.
2. After point 1 above, two pregnant (confirmed by diagnostic ultrasound and visualisation of fetal heartbeats) healthy ewes were vaccinated in the same way as in Point 1, with the same monitoring and recording measures, but also including a further ultrasound pregnancy examination after 7 days to confirm the presence of live fetuses.
3. Twenty‐one days after point 2, these same two ewes were revaccinated in the same manner as in 1 and 2 above, with the same monitoring and pregnancy ultrasound scanning as in point 2.

### Trial design

**FIGURE 1:**
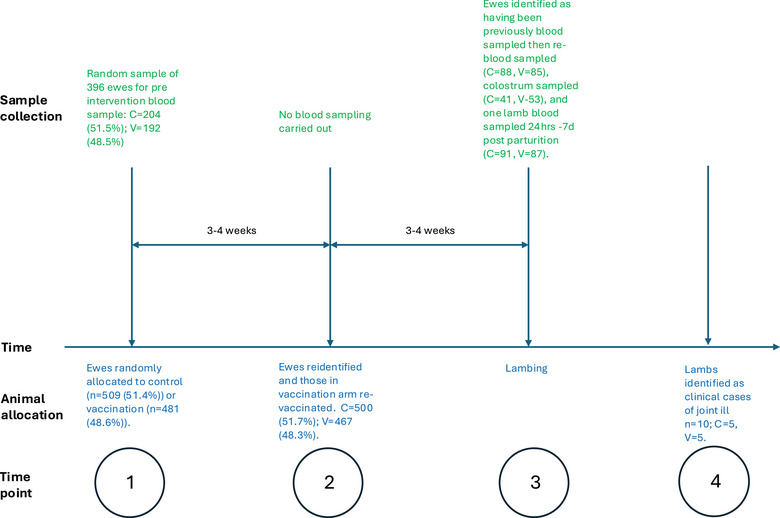
Visual description of the animal flow through the study, including trial arm allocation, blood and colostrum sampling and clinical case identification. C, control arm; V, vaccination arm.

The trial was conducted as a simple, blinded, randomised controlled trial and was carried out in 2022–2023. The whole flock was included in the study, with ewes randomly allocated to either the control arm or the vaccination arm using random numbers (Figure 1). Ewes were recruited to the study in mid‐pregnancy (time point 1, Figure 1) and identified by their unique ear tag number, which was then recorded against their random number, allocating them to a trial arm. At entry, background characteristics were recorded (by JA, LJ, NA and KK), including breed, age and body condition score. Ewes allocated to the vaccination arm were given the vaccine, administered by JA. No placebo or other intervention was given to the control ewes. After 3–4 weeks, the ewes were all reidentified and rerecorded, with ewes in the vaccination arm revaccinated, again by JA. From this point forward, the researchers and farmers were blinded to the trial arm the ewes were allocated to. The vaccinated and control ewes were managed homogenously within the two breed groups, with identification of the trial arm only revealed during the analysis stage at the end of the trial.

After each vaccination session, the flock was monitored by visual inspection for a minimum of 30 minutes by the veterinarian for any immediate adverse effects. Thereafter, the flock was returned to the care of the farmer to monitor for any adverse signs, including inappetence, self‐isolation, lameness, death or abortion, with subsequent referral to the veterinarian. Close contact was maintained between the farmer and veterinarian throughout.

A subset of 200 animals from each group was randomly selected to be blood sampled (by JA and JD) pre‐vaccination (time point 1, Figure 1) and at lambing, at least 3 weeks after the full vaccination course (time point 3, Figure 1). Sample size calculations were carried out based on unpublished pilot ELISA data from 26 ewes from a Norwegian study. Based on a power of 80%, a significance level of 95% and an effect size of 0.3, 175 sheep were needed per group (control and vaccinated). The increase in the number to 200 sheep per group was to allow for errors and loss of sheep to follow up.

For this subset of ewes, a colostrum sample was collected as soon as possible after lambing (within 24 hours) (by NA), and a blood sample from one of their lambs was taken between 24 hours and 7 days after lambing (by JA and JD). All samples were tested for SDSD‐specific antibodies using a validated SDSD ELISA assay (as described below). The colostrum samples were also analysed at the point of collection using a Brix refractometer. The lamb blood samples were also analysed for their total protein concentration using a refractometer. Serum was obtained from all blood samples, and both sera and colostrum were stored at −20°C for ELISA assays. During the lambing period, any lambs displaying signs of joint‐ill were examined and a joint fluid sample taken from the swollen joints to test for SDSD by bacterial culture, with species confirmation by matrix‐assisted laser desorption ionisation time‐of‐flight mass spectrometry (Microflex, Bruker Daltronics).

An in‐house ELISA for SDSD, developed by the Moredun Research Institute, was used to determine SDSD antibody titres in sera.^13^ ELISA plates were coated with 100 µL/well of a clinical isolate of SDSD, inactivated in paraformaldehyde, diluted 1:20 in coating buffer (7.92% 0.2 M sodium carbonate, 17.1% 0.2 M sodium hydrogen carbonate, pH 9.6) and sealed overnight at 4°C. Plates were washed six times in PBS Tween‐20 (PBS‐T) before blocking with 100 µL/well of 5% donkey serum (Sigma‐Aldrich) in 1× PBS for 1 hour, sealed at room temperature. Plates were washed in PBS‐T twice before substrate addition. Sera from study animals were added to plates, and doubling dilutions were performed in sample diluent buffer (2% donkey serum in PBS‐T). All samples were tested in duplicate, with a set of positive standards included in each assay to allow comparison between plates. Standards consisted of doubling dilutions from 1:100 to 1:12,800 of sera with a known high SDSD antibody titre, provided by the Moredun Research Institute. Four wells with only 50 µL of sample diluent buffer were used as a ‘blank’ negative control. Plates were sealed and incubated for 1 hour at room temperature. Plates were washed a further six times with PBS‐T. SDSD‐specific antibodies were detected by incubating with 100 µL/well of horseradish peroxidase‐conjugated rabbit anti‐sheep (1:20,000) in blocking buffer for 1 hour, and then sealed at room temperature. Plates were washed a final six times with PBS‐T before the addition of 100 µL of 3,3′,5,5′‐tetramethylbenzidine to each well. Plates were left unsealed and in the dark for 15 minutes for the colour to develop. The reaction was halted with the addition of 20 µL of H_2_SO_4_, and optical densities were read at 450 nm in a Thermo Scientific MultiSkan FC (Thermo Fisher Scientific).

### Statistical analyses

Descriptive statistics were employed to explore the characteristics of the ewes enrolled in each arm of the study, with chi‐squared tests used to analyse any differences in characteristics between the two arms. A two‐sample test of proportions was used to investigate the proportion of sheep allocated to each arm of the trial compared to the hypothesised proportion of 0.5.

Histogram plots were used to explore the distribution of the ELISA data for each time point, and Shapiro–Wilk tests were used to assess normality. Due to the skewed nature of the ELISA values at each time point, a Kruskal–Wallis equality of populations rank test was used to compare the control and vaccinated populations.

For the control group, when comparing the serum ELISA values at time point 1 with their matched ELISA values obtained at lambing (time point 3), a sign‐rank test was used.

The null hypotheses tested were as follows:

Primary clinical outcome:

1. There would be no difference between the number of lambs with joint‐ill born to vaccinated and unvaccinated ewes.

Secondary outcomes:

2. At entry to the study (time point 1), there would be no difference between the baseline serum ELISA values for the ewes in the control group and those in the vaccinated group.
3. At lambing (time point 3), there would be no difference between the serum ELISA values for the vaccinated and control ewes.
4. At lambing (time point 3), there would be no difference between the colostrum ELISA values for the vaccinated and control ewes.
5. At lambing (time point 3), there would be no difference between the Brix percentage values for the colostrum samples from vaccinated and control ewes.
6. At lambing (time point 3), there would be no difference between the serum ELISA values for lambs born to vaccinated and control ewes.
7. At lambing (time point 3), there would be no difference between the serum total protein values for lambs born to vaccinated and control ewes.
8. At lambing (time point 3), there would be no difference between the serum ELISA values for the control ewes compared to these same ewes at baseline (time point 1).

*p*‐Values of 0.05 or greater were considered weak evidence of a difference, while smaller *p*‐values were considered strong evidence of a difference.

## RESULTS

### Participant flow

In total, 990 ewes were recruited into the study. Their characteristics are detailed in Table 1. Of the 990 ewes, 509 (51.4%) were enrolled as controls and 481 (48.6%) were vaccinated. A two‐sample test of proportions comparing these two proportions to a hypothesised proportion of 0.5 revealed no difference (*p* = 0.4). Chi‐squared tests of the proportions of sheep with a particular characteristic in each arm of the trial revealed significant differences for the variable ‘age’ (*p *< 0.001) (Table 1).

**TABLE 1:** Baseline characteristics of the ewes enrolled in the study.

Variable	Controls *n* (%)	Vaccinated *n* (%)	Statistical comparisons between groups
Group (*n* = 990)	509 (51.4)	481 (48.6)	*p* = 0.9^*^
Age (years) (*n* = 981)			
1	131 (53.3)	115 (46.8)	
2	45 (34.9)	84 (65.1)	
3	109 (49.8)	110 (50.2)	
4	61 (57.0)	46 (43.0)	
5	110 (50.0)	110 (50.0)	
6	51 (85.0)	9 (15.0)	*p* < 0.001^†^
Breed (*n* = 990)			
Crossbred	323 (51.9)	299 (48.1)	
Welsh	186 (50.5)	182 (49.5)	*p* = 0.7^†^
Body condition score (*n* = 365)			
1	0 (0.0)	1 (100)	
1.5	5 (100)	0 (0.0)	
2	106 (54.4)	89 (45.6)	
2.5	47 (50.0)	47 (50.0)	
3	34 (49.3)	35 (50.7)	
3.5	0 (0.0)	1 (100)	*p* = 0.2^†^
Litter size (*n* = 461)			
1	40 (47.1)	45 (52.9)	
2	173 (51.3)	164 (48.7)	
3	18 (46.2)	21 (53.9)	*p* = 0.7^†^

*Note*: The ^*^ relates to a *p* value generated by a one sample test of proportions and the ^†^ to a *p* value from a *Χ* ^2^ test.

Of the 990 ewes enrolled in the study, 967 remained in their allocated arm: 500 (51.7%) controls and 467 (48.3%) vaccinated. Fourteen ewes received just one dose of vaccine (first or second), and nine were lost to follow‐up. A two‐sample test of proportions comparing the proportion of ewes remaining in the control arm and vaccinated arm to a hypothesised proportion of 0.5 revealed no difference (*p* = 0.3).

Blood samples were obtained from a random sample of 396 ewes at enrolment, and for those in the vaccination arm, this was carried out before vaccination. Of those allocated to be blood sampled, 204 (51.5%) were unvaccinated and 192 (48.5%) were vaccinated (*p* = 0.9). At lambing, at least 3 weeks after the second vaccine administration, 178 ewes that had been originally blood sampled were reidentified and resampled. The majority of the ewes that were unavailable for resampling were in the Welsh group; it was found to be too difficult to catch them at pasture after lambing without risking mismothering of the lambs. Pairs of blood samples were then available for 88 (50.9%) control ewes and 85 (49.1%) vaccinated ewes.

Colostrum samples were obtained from 41 (43.6%) control ewes and 53 (56.4%) vaccinated ewes. Blood samples were also obtained from 91 (51.1%) lambs born to ewes in the control group and 87 (48.9%) lambs born to ewes in the vaccinated group.

### Vaccine safety

No vaccine‐associated adverse effects were noted, although there was a transient rise in body temperature 24 hours after vaccination.

### Outcomes

#### Primary outcome

**Null hypothesis 1**: There would be no difference between the number of lambs with joint‐ill born to vaccinated and unvaccinated ewes.

Ten lambs were diagnosed with joint‐ill (compared to approximately 70 the year before). Five of the lambs were from unvaccinated ewes and five were from vaccinated ewes (Table 2). Due to the small number of cases, analysis of associations with specific variables was not performed. The null hypothesis was not disproved.

**TABLE 2:** Characteristics of the lambs that developed joint‐ill.

Ewe ID	Group	Ewe age	Ewe breed	Litter size	Lamb sex	Pre‐vaccinated serum ELISA	At lambing serum ELISA	Colostrum ELISA	Lamb serum ELISA	Colostrum Brix (%)	Lamb total protein (g/dL)
7	Control	6	Crossbred	2	Male	519.3	1612.9	589.2	161.8	30	38
16	Control	5	Crossbred	2	Male	32.3	145.1	‐	160.2	30	‐
329	Control	5	Crossbred	2	Male	292.7	5.2	663.2	667.6	30	49
363	Control	2	Crossbred	2	Male	1276.0	937.0	370.8	835.5	29	47
385	Control	1	Crossbred	2	‐	‐	‐	‐	‐	‐	‐
96	Vaccinated	3	Crossbred	3	‐	‐	‐	‐	‐	30	‐
128	Vaccinated	3	Crossbred	2	Male	163.1	191.0	‐	321.7	‐	43
246	Vaccinated	5	Crossbred	2	‐	‐	‐	‐	‐	18	‐
12	Vaccinated	2	Crossbred	2	Female	466.2	524.7	‐	1340.8	30	42
342	Vaccinated	3	Crossbred	2	Female	474.2	467.4	428.6	55.5	19	28

#### Secondary outcomes (Table 3)

Histogram plots of the ELISA data demonstrated right‐skewed distributions for most of the analysis groups, and Shapiro–Wilk tests of normality indicated the data were not normally distributed. As such, non‐parametric tests were employed to make comparisons.

**Null hypothesis 2**: At entry to the study (time point 1), there would be no difference between the baseline serum ELISA values for the ewes in the control group and those in the vaccinated group.

At time point 1, the median baseline serum ELISA value for the control group (*n* = 88) was 422.5 (interquartile range [IQR]: 174.0–816.1), and for the vaccinated group (*n* = 85) it was 441.8 (IQR: 144.1–703.3). A Kruskal–Wallis equality of populations rank test demonstrated no significant difference between the two groups (*p* = 0.5). Null hypothesis 2 was not disproved.

**Null hypothesis 3**: At lambing (time point 3), there would be no difference between the serum ELISA values for the vaccinated and control ewes.

At time point 3, the median serum ELISA value for the control ewes was 571.0 (IQR: 191.7–1159.6), and for the vaccinated ewes it was 873.2 (IQR: 442.1–1532.0). A Kruskal–Wallis equality of populations rank test revealed the median for the vaccinated ewes to be greater than that of the unvaccinated ewes (*p* = 0.02). Accounting for the background pre‐vaccinated ELISA values by subtracting them from the post‐vaccinated ELISA values, the median for the control ewes was 156.0 (IQR: −215.4 to 657.1) and for the vaccinated ewes was 393.7 (IQR: 58.4–946.1). A Kruskal–Wallis equality of populations rank test revealed the median for the vaccinated ewes to be greater than that for the unvaccinated (*p* = 0.02). Null hypothesis 3 was disproved.

**Null hypothesis 4**: At lambing (time point 3), there would be no difference between the colostrum ELISA values for the vaccinated and control ewes.

At time point 3, the median ELISA value for the colostrum samples from the control group (*n* = 41) was 1010.4 (IQR: 589.2–2243.4), and for the vaccinated group (*n* = 53) it was 1134.1 (428.6–1883.3). A Kruskal–Wallis equality of populations rank test revealed no difference between the two groups (*p* = 0.8). Null hypothesis 4 was not disproved.

**Null hypothesis 5**: At lambing (time point 3), there would be no difference between the Brix percentage values for the colostrum samples from vaccinated and control ewes.

At time point 3, the median Brix percentage for colostrum samples from the control ewes (*n* = 211) was 29% (IQR: 24–30%), and for the vaccinated ewes (*n* = 217) it was 30% (IQR: 25–30%). A Kruskal–Wallis equality of populations rank test revealed no difference between the two groups (*p* = 0.1). Null hypothesis 5 was not disproved.

**Null hypothesis 6**: At lambing (time point 3), there would be no difference between the serum ELISA values for lambs born to vaccinated and control ewes.

At time point 3, the median serum ELISA value for lambs from the control group (*n* = 91) was 662.3 (IQR: 211.3–1469.2), and for the vaccinated group (*n* = 87) it was 1171.9 (IQR: 372.3–1903.5). A Kruskal–Wallis equality of populations rank test revealed the median for the lambs born to vaccinated ewes to be greater than that for the lambs born to unvaccinated ewes (*p* = 0.02). Null hypothesis 6 was disproved.

**Null hypothesis 7**: At lambing (time point 3), there would be no difference between the serum total protein values for lambs born to vaccinated and control ewes.

At time point 3, the median serum total protein concentration for lambs born to ewes in the control group (*n* = 90) was 6.9 g/dL (IQR: 6.1–7.7), and for the vaccinated group (*n* = 87) it was 6.9 g/dL (IQR: 6.0–7.7). A Kruskal–Wallis equality of populations rank test revealed no difference between the two groups (*p* = 0.7). Null hypothesis 7 was not disproved.

**Null hypothesis 8**: At lambing (time point 3), there would be no difference between the serum ELISA values for the control ewes compared to these same ewes at baseline (time point 1).

At time point 3, for the control ewes only (*n* = 88), a signed‐rank test comparing the ELISA values at this time point with those obtained at time point 1 revealed that the overall values had increased, (*p* = 0.02). As detailed above and in Table 3, the median value for the control ewes at the pre‐vaccination time point was 422.5 (174.0–816.1) and at lambing was 571.0 (191.7–1159.6). Null hypothesis 8 was disproved.

**TABLE 3:** ELISA values and the results of statistical tests comparing the control and vaccinated groups.

	Control group median (IQR)	Vaccinated group median (IQR)	Kruskal–Wallis test result
Pre‐vaccine serum ELISA (controls *n* = 88; vaccinated *n* = 85) (Time point 1)	422.5 (174.0–816.1)^a^	441.8 (144.1–703.3)	*p* = 0.5
At lambing serum ELISA (controls *n* = 88; vaccinated *n* = 85) (Time point 3)	571.0 (191.7–1159.6)^a^	873.2 (442.1–1532.0)	*p* = 0.02
At lambing serum ELISA minus pre‐vaccine ELISA (controls *n* = 88; vaccinated *n* = 85) (Time point 3)	156.0 (−215.4 to 657.1)	393.7 (58.4–946.1)	*p* = 0.02
Colostrum ELISA (controls *n* = 41; vaccinated *n* = 53) (Time point 3)	1010.4 (589.2–2243.4)	1134.1 (428.6–1883.3)	*p* = 0.8
Lamb serum ELISA (controls *n* = 91; vaccinated *n* = 87) (Time point 3)	662.3 (211.3–1469.2)	1171.9 (372.3–1903.5)	*p* = 0.02

^a^ These two values were compared using a Wilcoxon signed‐rank test, resulting in a *p* value of 0.02.

## DISCUSSION

### Study design and limitations

In general, randomisation was effective, with statistically equal proportions in each arm of the trial at each subcategory of analysis. For the variable ‘age’, some differences were found between the proportion of sheep allocated to the control and vaccination arms for those aged 2, 4 and 6 years; however, it was considered unlikely to affect the results.

The ELISA data were limited mainly to crossbred ewes due to the practical challenges of trying to follow up Welsh ewes lambed outside without causing harm. This considerably reduced the number of samples analysed and will have reduced the precision of the estimates produced.

The ELISA used, while specific to SDSD IgG, has not been correlated with a specific concentration of IgG. As such, while comparisons can be made, translation of a specific ELISA value to a specific concentration of IgG cannot yet be made.

### Generalisability

Due to the real‐world context in which this study was carried out, similar results could be expected on other farms. Using a whole‐flock vaccination approach, or group (cluster) randomisation, as opposed to individual randomisation, would likely have led to enhanced effects due to the expectation of a herd immunity benefit at the flock or group level, as well as potentially reduced environmental contamination.

### Safety

In the safety study, the only observed physiological effect of the vaccine on the ewes, other than the positive serological effects, was a transient temperature rise. During the main field trial, no other adverse events (e.g., abortions, inappetence or death) were noted. As such, the vaccine was considered safe.

### Interpretation of results

The vaccine did not reduce the number of lambs with joint‐ill born to vaccinated ewes compared with unvaccinated ewes, with equal numbers of cases (*n* = 5) in both arms of the trial. However, compared to previous years, the overall number of cases (*n* = 10) was considerably lower than expected, with the farmer previously reporting approximately 70 cases in a season. This perceived reduction in the overall number of cases observed could be due to chance or possibly as a result of an overall reduction in the amount of SDSD shed into the environment from the vaccinated ewes. Studies using cluster randomisation and environmental sampling with quantification of the amount of SDSD in the environment could help unravel this effect.

The pre‐vaccination serum ELISA values varied widely. This is perhaps expected in an endemic flock, with some ewes generating more IgG than others depending on their exposure, the timing of exposure and their individual immunological response.

At lambing, there was an increase in the ELISA values for both the control and vaccinated ewes, with a statistically greater increase for the vaccinated ewes compared to the control ewes. This was most likely interpreted as a vaccination response. It is not yet known what IgG concentration (and corresponding ELISA value) would be protective or indicate a satisfactory response, with in vivo challenge studies required to investigate this.

For the control ewes, this increase in ELISA values was unexpected. A sign‐rank test indicated a low probability that this increase occurred by chance, and therefore, this change is a result of one or more factors. The ewes were managed close to one another for lambing (inside and outside), rendering the possibility of environmental contamination by infected ewes more likely and increasing the opportunity for ewes to be exposed to large volumes of SDSD. In other infectious disease scenarios, it is well recognised that infected ewes undergo a form of immune suppression associated with the periparturient period, resulting in an increased susceptibility to disease and infection. In the case of gastrointestinal nematode infection, this is termed the ‘periparturient rise’ due to the observed increase in faecal nematode egg output.^14^ It is possible that, due to the ewes being tested at the point of lambing, some of the ewes experienced reduced immunity to SDSD with a consequent increase in shedding of SDSD into the environment—a form of periparturient rise. Further investigation would be useful to understand the causes behind this finding.

There was no difference observed in the ELISA values for the colostrum samples from control and vaccinated ewes. In this field study, the exact timing of lambing was uncontrolled, and the exact timing from birth to the collection of the colostrum sample varied. Because of this, it was possible that some lambs may have sucked colostrum from the ewe before sampling, reducing colostrum density by the time a sample was collected. This theory was supported by the fact that lambs born to vaccinated ewes had a correspondingly elevated serum ELISA value compared to those born to control ewes, reflecting the vaccine response seen in the ewe samples at lambing. To investigate the possibility that the elevated serum ELISA values in the lambs born to vaccinated ewes was somehow a result of a systematic bias in lambs born to vaccinated ewes having received a greater volume of colostrum, or having received it more quickly, a comparison of the serum total protein values was carried out, with no difference observed between lambs born to vaccinated ewes and those born to control ewes. As such, as with all vaccinations requiring successful maternal transfer, the successful use of a vaccine against joint‐ill will ultimately depend not only on the technical aspects of the vaccine and administration but also on the ability of the lambs to obtain a sufficient volume of colostrum of sufficient density in a short period after birth.

## CONCLUSIONS

In this study, the vaccine did not reduce the number of lambs with joint‐ill born to vaccinated ewes compared with unvaccinated ewes. However, vaccinating ewes did have a positive effect on their serum SDSD ELISA values and those of their lambs. Further work is needed to explore the herd immunity effects of the vaccine and the concentration of SDSD‐specific IgG necessary to affect protection from SDSD infection.

## AUTHOR CONTRIBUTIONS

Joseph W. Angell and Jennifer S. Duncan conceived the study and designed and executed it. Naseem Ahmed collected data on the farm and carried out laboratory analyses. Louise P. Jackson and Konstantina Kasiora carried out laboratory analyses, and Keith Ballingall provided laboratory oversight and technical support. Joseph W. Angell wrote the manuscript, and all authors reviewed and edited the manuscript and provided intellectual input.

## CONFLICT OF INTEREST STATEMENT

The authors declare they have no conflicts of interest.

## ETHICS STATEMENT

The study was approved under the Animals (Scientific Procedures) Act 1986, Home Office Project License Number PP1744936. Ethical approval for the study was obtained from the University of Liverpool, number VREC 1242. The vaccine trial was licensed by the Veterinary Medicines Directorate with Animal Test Certificate ATC/B 56268/0002. Written informed consent was obtained from the farmer.

## Data Availability

The data that support the findings of this study are available from the corresponding author upon reasonable request.
